# Improving Palliative Care in Residential Aged Care Using Telehealth: Protocol for a Realist Process Evaluation Embedded in a Stepped-Wedge Cluster Randomized Controlled Trial

**DOI:** 10.2196/68332

**Published:** 2025-10-20

**Authors:** Kayla Lock, Anita M Y Goh, Katrin Gerber, Joanne Tropea, Kirsten Moore, Wen Kwang Lim

**Affiliations:** 1 Department of Medicine - Royal Melbourne Hospital Faculty of Medicine, Dentistry, and Health Sciences University of Melbourne Melbourne Australia; 2 National Ageing Research Institute Melbourne Australia; 3 Department of Psychiatry Faculty of Medicine, Dentistry, and Health Sciences University of Melbourne Melbourne Australia; 4 School of Media and Communication RMIT University Melbourne Australia; 5 Melbourne School of Psychological Sciences Faculty of Medicine, Dentistry, and Health Sciences The University of Melbourne Melbourne Australia; 6 Department of Aged Care Royal Melbourne Hospital Melbourne Australia

**Keywords:** aged care, end of life care, implementation, palliative care, process evaluation, realist evaluation, residential aged care, telehealth

## Abstract

**Background:**

This study describes the protocol for a realist process evaluation of IMPART (Improving Palliative Care in Residential Aged Care Using Telehealth), to be trialed through a pragmatic stepped-wedge cluster randomized controlled trial in Australia. IMPART consists of 2 key intervention activities: specialist palliative support provided through telehealth and tailored staff education.

**Objective:**

The aims of the realist process evaluation are to (1) identify and explore the contexts and mechanisms that enable or hinder the implementation of the IMPART intervention, and (2) develop and refine a program theory to determine whether and how successful implementation of IMPART can be facilitated.

**Methods:**

We will conduct this process evaluation in 3 phases, guided by a realist framework. First, to hypothesize an initial program theory, we will review trial documentation and literature to determine how IMPART is expected to work and identify the barriers and facilitators likely to influence implementation. To test this theory in the second phase, a case study methodology will draw on multiple data sources (qualitative and quantitative) from 10 participating residential aged care facilities. These include interviews with staff involved in implementation, data on staff engagement with training, program documentation, activity logs, action plans, and facility information. In the final phase, program theories developed from the case studies will be refined through consultation with the IMPART research team. This will inform the development of a refined program theory that provides key information about what works, for whom, how, and in what circumstances in the implementation of interventions aiming to improve palliative care in residential aged care.

**Results:**

This study was reviewed and approved by the Royal Melbourne Hospital Human Research Ethics Committee. The randomized controlled trial commenced in May 2023, with completion anticipated in November 2025. Funding began in January 2022. Data for the realist process evaluation will be collected between May 2023 and February 2026. As of October 2025, a total of 61 interviews have been completed. Data analysis is ongoing, and a publication describing the results will be prepared in 2026.

**Conclusions:**

Applying a realist framework to explore process outcomes allows for an in-depth inquiry into what works, for whom, how, and in what circumstances in the implementation of complex interventions aiming to improve palliative care in residential aged care. This realist process evaluation has the potential to provide transferable, context-specific findings that can support the development of meaningful policy and accelerate practice change.

**Trial Registration:**

Australian New Zealand Clinical Trials Registry ACTRN12622000760774; https://tinyurl.com/2uv34unr

**International Registered Report Identifier (IRRID):**

DERR1-10.2196/68332

## Introduction

### Overview

In Australia, residential aged care facilities are the second most common place of death after hospitals [1]. Residential aged care, also known as nursing homes or long-term care, provides accommodation and 24-hour care for older people who can no longer live independently at home. In 2022-2023, 84% of exits from permanent residential aged care facilities were due to death, with an average length of stay of 21 months [2]. Yet, in Australian residential aged care facilities, a lack of clarity about residents’ end-of-life care preferences is widely reported [3,4]. The quality of end-of-life care provided across the residential aged care sector also varies [5]. Studies suggest that insufficient staff capacity and resources [6], lack of staff confidence and knowledge [7-9], and residents’ complex needs [10] contribute to this variation in quality of care. End-of-life care discussions are often avoided by residents, families, and staff in residential aged care, which can lead to suboptimal decision-making during medical crises [11,12]. Residents of residential aged care facilities are more likely to present to hospital emergency departments than older people living in the community [13,14]. These admissions, often considered avoidable, can result in the resident dying in hospital when appropriate care could have been provided at the residential aged care facility with the necessary supports in place [14,15]. More effective channels of communication and documentation of care preferences are needed.

Palliative care is an approach that focuses on improving the quality of life of patients and their families who are at the end of life through “the prevention and relief of suffering of any kind—physical, psychological, social, or spiritual” [16]. Palliative care should be considered the core business of aged care in Australia [17,18]. Accordingly, the Australian Royal Commission into Aged Care Quality and Safety recommended that palliative care be identified as a core competency to be included in the training and certification of residential aged care staff and that access to timely provision of specialist palliative care and other relevant specialists be expanded for residential aged care facilities [18]. In 2021-22, approximately 246,000 Australians lived in residential aged care facilities, with 2% appraised under the Aged Care Funding Instrument as needing palliative care services, which were often available only in the last days of life [1,17]. However, Palliative Care Australia argue that palliative care services in residential aged care are underfunded and underserviced [18]. The Comprehensive Palliative Care in Aged Care measure (2018-2024) introduced by the Australian Government, aimed to strengthen national efforts to improve access to quality palliative care in residential aged care. Projects supported by the measure found that improved communication channels with palliative care specialists were facilitated through telehealth use [19,20].

Telehealth, as described by the International Organization for Standardization, is the “use of telecommunication techniques for the purpose of providing telemedicine, medical education, and health education over a distance” [21]. This may occur through communication services such as a telephone, video, or messaging platforms [21]. Demand for telehealth has greatly increased due to the COVID-19 pandemic [22]. Telehealth has been reported to have the potential to improve access and clinical outcomes and reduce the cost of service delivery [18,23]. The Australian Government Digital Health Strategy 2023-2028 has consequently focused on widening access to telehealth services [24]. Individual-, interpersonal-, community-, and societal-level factors influence the successful uptake of health technologies such as telehealth [25]. A 2019 systematic review found that the benefits of using telehealth for palliative care, such as reduced need for emergency care, are often described without being adequately evaluated [26]. The review identified only one study that was set in residential aged care.

Since COVID-19 several studies have explored the use of telehealth to improve palliative care in residential aged care. Telehealth uptake has led to increased discussion and documentation of goals of care, improved symptom management, and decrease use of acute care use [27-29]. Participants were generally accepting of and willing to use telehealth to connect with palliative care specialists, particularly those living in rural areas [20,27,28,30,31]. Persistent barriers, however, included technical and connectivity issues, financial burden to the facility, concerns about privacy, logistics, and inflexibility of specialists [27,29,30]. The successful implementation and sustainability of telehealth-enabled palliative care interventions in residential aged care depend on policy commitment, clear practice recommendations, and financial support, with no “one size fits all” approach [20,29]. To support this, urgent attention is needed to evaluate intervention effectiveness and implementation outcomes [27,29,31].

### The IMPART (Improving Palliative Care in Residential Aged Care Using Telehealth) Intervention

In 2020, the National Health and Medical Research Council, on the advice of the Australian Health Ministers’ Advisory Council Working Committee, released a targeted call for research into end-of-life care, citing limited evidence to inform policy and practice [32]. In response to this call, and to support residential aged care facilities in improving palliative care, the IMPART (Improving Palliative Care in Residential Aged Care Using Telehealth) intervention was developed. IMPART aims to reduce avoidable hospitalization at the end of life by engaging specialist support provided through telehealth and tailored staff education. Its intended outcomes are to enable timely end-of-life discussions, improve documentation of care preferences and, therefore, enable preference-based care, reduce unplanned hospitalization, and improve residents’ quality of end-of-life care. The program is being tested in a 2.5-year pragmatic, stepped-wedge, cluster randomized controlled trial (RCT), involving 10 residential aged care facilities located in metropolitan regions of Melbourne, Australia, along with 3 residential in-reach services accessed through telehealth. Residential in-reach services provide acute care within residential aged care facilities to reduce the need for hospitalization of residents [14]. This realist process evaluation is embedded within the IMPART trial. The trial consists of five 6-month waves, in which 2 new residential aged care facilities will start the active phase of the IMPART intervention during every wave in a randomized order. The trial schedule is displayed in Table 1. The full trial protocol was published in July 2025 [33].

**Table 1:** Overview of IMPART (Improving Palliative Care in Residential Aged Care Using Telehealth) intervention roll-out. Each block represents a 6-month period. ✓=Data collection point. Intervention=facilities actively receiving the IMPART intervention during that block. Control/waiting=facilities yet to receive the intervention.

Intervention roll-out (2 facilities per step)	Year 1		Year 2		Year 3	
	Block 0	Block 1	Block 2	Block 3	Block 4	Block 5
Roll-out 1		✓				
Roll-out 2			✓			
Roll-out 3				✓		
Roll-out 4					✓	
Roll-out 5						✓

### Five Core Components of the IMPART Intervention

The IMPART intervention comprises 5 core components: component 1 involves forming a planning ahead team to guide implementation, component 2 focuses on conducting a local needs analysis; component 3 entails a facilitated workshop to co-design an action plan, component 4 provides targeted online training and goals-of-care education, and component 5 engages local specialists to provide telehealth support and monitor progress over 6 months.

#### Component 1

In month 1 of the intervention, a planning ahead team will be formed at each facility, made up of 2-3 staff (nurses, clinical care coordinators, managers, or anyone else with a passion for palliative care) who will champion the program, along with general practitioners who regularly attend the participating facilities. The planning ahead team will be provided with an intervention manual that will guide them through implementation for the 6-month period.

#### Component 2

Additionally, in month 1, the planning ahead team, with facilitation as required from the research team, will undertake a local needs analysis to identify areas for improvement in end-of-life care discussions, documentation, and provision. This involves a short survey assessing the planning ahead team’s confidence in providing, discussing, and planning for end-of-life care, a postdeath file audit of end-of-life care documentation, and an audit of advance care planning documentation.

#### Component 3

In month 2, the research team will facilitate a workshop with the planning ahead team to present findings from the local needs analysis, highlighting strengths and challenges in current end-of-life care practice. The workshop allows the planning ahead teams to meet and establish communication channels between the residential aged care facility and in-reach teams. At the workshop, an action plan will be co-designed to tailor the intervention to meet their needs as identified during the session. The action plan will be implemented and monitored in months 3-6.

#### Component 4

Residential aged care facility staff will be given access to an online training package IMPETUS-D (Improving Palliative care Education and Training Using Simulation in Dementia) [6]. IMPETUS-D includes 11 modules containing video simulations, each taking approximately 15-30 minutes to complete. The IMPART intervention aims to tailor the application of the IMPETUS-D modules, allowing the planning ahead teams to incorporate the modules most relevant to them into their action plan. A 90-minute goals-of-care online training session, provided by aged care specialists, will also be arranged for each facility.

#### Component 5

Local palliative and aged care specialists will be engaged as facilitators to provide telehealth in-reach and participate in the planning ahead teams to support residential aged care facility staff in achieving the goals set out in their action plans. The aim is to foster rapid communication between the residential aged care facilities and in-reach teams through telehealth to promote sustainable collaborative working relationships beyond the completion of the study. The planning ahead teams will aim to meet fortnightly during months 3-6.

### Realist Process Evaluation of Complex Interventions

According to the Medical Research Council, an intervention may be considered complex if it fits one or more of the following categories: contains multiple components, targets a range of behaviors, permits a level of flexibility, or requires a level of expertise and skills from those delivering and receiving the intervention [34]. As such, IMPART can be regarded as a complex intervention. Many complex interventions targeting residential aged care have demonstrated potential to reduce unplanned hospitalizations [35,36]. A systematic review found that more than one intervention is likely needed to impact hospitalization of residential aged care residents, with those targeting advance care planning or goals-of-care setting being the most effective [36]. Yet, if these interventions work and achieve their desired outcomes of reducing unplanned hospitalizations and inducing practice change in staff, how and in what circumstances is implementation successful? With limited evidence on how telehealth-enabled palliative care interventions function in residential aged care, there is a need to uncover causal linkages to reproduce the results and inform national strategies.

The Medical Research Council guidelines for evaluating complex interventions highlight the importance of investigating the interactions between the intervention and its context to identify mechanisms of change, “where mechanisms are the causal links between intervention components and outcomes” [34]. This is consistent with a realist evaluation framework which seeks to form context–mechanisms–outcomes configurations (CMOC), or context+mechanisms=outcomes, in order to understand for whom, how, and in what circumstances an intervention works [37,38]. Context is defined as the relational and dynamic factors not formally part of the intervention but having impact, for example, the physical and social environment, existing policy, cultural norms, and values [39]. This protocol describes a realist process evaluation of IMPART, conceptualized as a theory-informed approach that aims to strengthen the impact of evaluation on policy and practice [37]. A realist process evaluation thus answers questions relating to fidelity and quality of implementation, mechanisms of change, and context. Further, the realist understanding of fidelity investigates when and how adaptations are made to the RCT protocol and the reasoning behind such changes [40]. Successful implementation is more likely to be reproduced whereby these questions are answered.

The compatibility of realist evaluation and RCTs has been rigorously debated [41-43]. Highlighted in these discussions are the fundamental differences in ontological and epistemological perspectives, with realism distinguished by its understanding of causation [40]. The role of the realist evaluator is “to explain how and why programs or policies cause their various outcomes in different sets of circumstances” [40]. In contrast, post-positivist logic underpins RCTs, conflicting with the realist view that there are no “final” truths or knowledge [40]. This protocol takes guidance from the realist process evaluation undertaken by Rycroft-Malone et al [43]. We do not consider this to be a “realist” RCT, but an RCT involving a process evaluation that is realist-informed. Each residential aged care facility will be evaluated as a case study to uncover the different mechanisms operating in different contexts, generating various outcomes. By embedding a realist process evaluation within the IMPART RCT, we can go beyond describing barriers and enablers to implementation and uncover causal linkages that have not been identified in previous studies evaluating telehealth-enabled palliative care interventions.

### Aims

The realist process evaluation has 2 aims. Aim 1 is to identify and explore the contexts and mechanisms that enable or hinder the implementation of the IMPART intervention, designed to improve palliative care in residential aged care using telehealth. This aim seeks to uncover the mechanisms inherent in successful implementation, understand how success is defined, and identify in what contexts and circumstances these mechanisms trigger success. Aim 2 is to develop and refine a program theory to optimize any future implementation of IMPART and similar interventions to improve palliative care in residential aged care. The primary research question seeks to answer what works, for whom, how, and in what circumstances in the implementation of the IMPART intervention. Subquestions address key implementation outcomes relating to intervention adoption, implementation fidelity, and protocol adaptations.

## Methods

### Ethical Considerations

This project was reviewed and approved by the Royal Melbourne Hospital Human Research Ethics Committee, protocol version 6 (HREC/84300/MH-2022). Original approval was granted on May 19, 2022. Participants will receive an invitation and consent form before the interview and may ask questions prior to providing written and verbal consent. Interviews will be audio-recorded (in-person, online, or by phone) based on participant preference. Each participant will receive an AUD $50 honorarium (approximately US $33), and all identifying details will be removed from transcripts to ensure anonymity. All data collected will be stored securely in a locked filing cabinet or under strict password protection in accordance with National Health and Medical Research Council requirements. Data will be deidentified by assigning participant codes. Only the research team will have access to the data. After analysis is completed, data will be retained for 7 years and then disposed of confidentially. Data will be used only for the current project.

### Study Design

**Figure 1:**
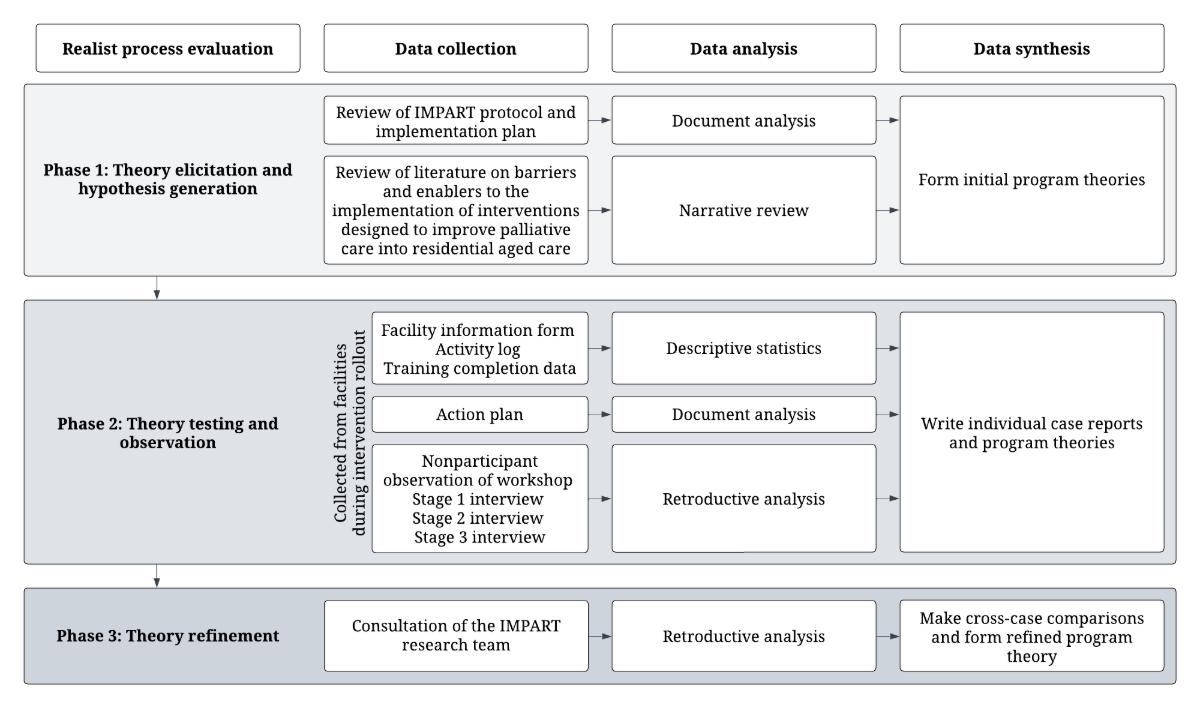
Overview of the IMPART (Improving Palliative Care in Residential Aged Care Using Telehealth) realist process evaluation.

This realist process evaluation involves three key phases conducted iteratively: (1) theory elicitation and hypothesis generation, (2) theory testing and observation, and (3) theory refining [43,44]. Data collection and analysis will occur concurrently with the IMPART trial during the roll-out of the active intervention at participating residential aged care facilities, cycling through the hypothesizing and testing of causal pathways [44,45]. Figure 1 provides an overview of the realist process evaluation. To ensure the study adheres to the preferred quality and reporting standards for realist evaluations, guidance will be taken from the Realist and Meta-narrative Evidence Syntheses: Evolving Standards (RAMESES) [40].

### Data Collection and Analysis

#### Phase 1: Theory Elicitation and Hypothesis Generation

A realist approach first identifies the relevant intervention theories and asks, what is suspected to cause what to happen? RAMESES suggests that there are many ways to identify an initial “rough” program theory which presents a significant challenge to those new to the realist approach [40,46]. Flynn et al [46] recommend researchers “live within their means” to mitigate extensive use of time and resources, noting that generating an initial program theory is just the first phase of a larger funded project. To generate a hypothesis and understand how the researchers intend for IMPART to work and why, a document analysis of the IMPART protocol and implementation plan was conducted. The content of the protocol and implementation plan was coded inductively with the following categories: implementation strategies, intervention activities, mechanisms, implementation outcomes, service outcomes, and resident outcomes.

To identify barriers and enablers likely to impact the implementation of the IMPART intervention, a narrative review was undertaken. Peer-reviewed literature published in English after 2000 was included. MEDLINE was searched using key terms such as “residential aged care,” “palliative care,” and “implementation.” Specialist journals (eg, Implementation science) and reference lists of identified studies were also hand searched. Articles were prioritized according to relevance and recency to increase significance of the findings to the current residential aged care setting. Literature was coded inductively using the Consolidated Framework for Implementation Research (CFIR) [47] categories: intervention characteristics, outer setting, inner setting, characteristics of individuals, and implementation process. Those determinants that reoccur most frequently were noted.

The results of the document analysis and narrative review supported the development of initial program theories and a coding framework to be used in the theory-testing phase. To ensure that the IMPART implementation strategies (how the intervention activities are being implemented) are clearly defined and distinguishable from the intervention activities (what is being implemented), guidance was taken from the Expert Recommendations for Implementing Change (ERIC) [48,49] and Proctor et al [50,51]. A total of 9 initial program theories were generated linking Context–Implementation Strategy–Mechanism–Implementation Outcome (Multimedia Appendix 1). The hypothesized mechanisms that will be tested draw on 3 established middle-range theories (Textbox 1). First, the Capability, Opportunity, and Motivation inform Behavior change (COM-B) model of behavior change is expected to influence training uptake [52-54]. Second, it is hypothesized that adoption of telehealth can be explained using the diffusion of innovation framework [55,56]. Finally, the 4 Normalization Process Theory constructs identified as mechanisms by May et al [57-59] are expected to inform how IMPART, as a complex intervention, is expected to work.

**Theories informing hypothesized mechanisms.**

**Capability, Opportunity, and Motivation inform Behavior change (COM-B)**

- If champions have capability and opportunity to engage with the IMPART (Improving Palliative Care in Residential Aged Care Using Telehealth) intervention, in addition to demonstrated motivation to improve palliative care practice, then uptake of the online training (IMPETUS-D [Improving Palliative care Education and Training Using Simulation in Dementia] and goals-of-care workshop) is more likely.

**Diffusion of innovation**

- If champions perceive specialist telehealth support to have potential benefits for residents, their families, and staff, then they will advocate for and act as early adopters.

**Normalization process theory**

- Coherence: If the planning ahead team is able to make sense of IMPART and its components through reflection and evaluation (needs analysis, IMPART workshop, and action planning), then they will differentiate the intervention from current ways of working and form collective agreement about the intervention purpose and actions required to achieve their goals.
- Cognitive participation: If IMPART is legitimized through the provision of additional funding and formal commitments from the research team, In-Reach service, and residential aged care facility, then there will be continued support to meet those commitments.
- Collective action: If formation of the planning ahead team and external facilitation allows champions to share the workload of implementation, then they will be able to mitigate staff changes and increase trust between services, which grants the In-Reach team membership to the residential aged care facility.
- Reflexive monitoring: If champions implement and monitor their action plans, then they will appraise the intervention through attainment of their tailored goals and the extent in which they meet the specific needs of the residential aged care facility.

#### Phase 2: Theory Testing and Observation

A case study methodology will test the 9 initial program theories during the trial of the IMPART intervention. Multiple qualitative and quantitative data sources will be triangulated [60,61]. This methodology is “an empirical inquiry that investigates a contemporary phenomenon in depth and within its real-life context, especially when the boundaries between phenomena and context are not clearly evident” [61]. The case study approach is appropriate for a realist evaluation and the evaluation of complex interventions, as it applies theories and methods in a similar tradition [62]. This methodology supports the analysis of the phenomenon (the IMPART intervention) in context to uncover the mechanisms that trigger specific outcomes [62] in the cases (the participating residential aged care facilities; n=10).

**Figure 2:**
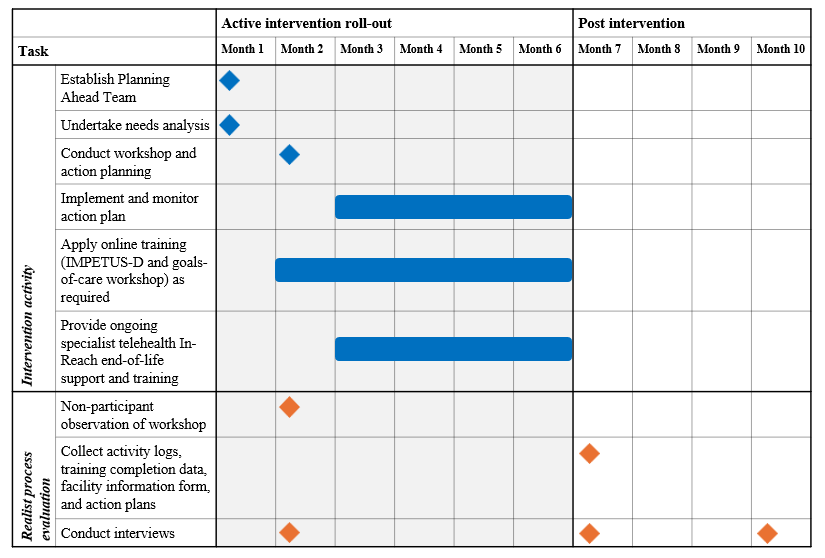
Timeline of IMPART (Improving Palliative Care in Residential Aged Care Using Telehealth) components. IMPETUS-D: Improving Palliative care Education and Training Using Simulation in Dementia.

The researcher will have direct access to the case to ensure a cooperative working relationship and that the study is of interest to the participants [63]. This will increase the likelihood that the data gathered are informative and will answer the research questions. The RAMESES guidelines recommend that cases are examined to the point of saturation, “checking that the patterns of success and failure, intended and unintended outcomes are consistent with the theory” [64]. Any new theories that emerge will be tested in further cases. The timeline of IMPART components and how they correspond to the realist process evaluation is displayed in Figure 2. How the data to be collected align with the initial program theories is available in Multimedia Appendix 1.

### Data Collection

#### Qualitative Data

Nonparticipant observation will be conducted at the IMPART workshops in month 2 of the intervention. The workshops will be conducted online and recorded. Before the workshop the planning ahead team will be provided detailed information about their involvement through a preintervention meeting and manual. Their consent to participation in the planning ahead team will be implied by attendance at the workshop. Participants will be notified that the workshop is being recorded and observed. No identifiable information or direct quotes will be collected during the observation; therefore, informed consent will not be collected from participants. Up to 10 workshops will be observed. The data collected will be used to explore whether residential aged care facility and in-reach staff recruited to implement the intervention have been engaged as intended and the extent to which participants agree that the intervention is able to fulfil a need and is appropriate. To guide data collection, Spradley’s [65] 9 dimensions of observation will be used. The dimensions cover the physical layout and objects present, the range of people involved, actions they undertake, the activities that take place and their sequencing, the goals that people are trying to achieve, and feelings expressed [65].

Upon completion of the intervention, action plans will be reviewed to assess whether the planning ahead teams were able to successfully implement the planned actions and whether the intervention addressed a need. Data from action plans will be used to assess implementation fidelity at participating facilities as well as to provide context. Data to be extracted and categorized will include the number of goals set and perceptions of attainment, the problem the goal is seeking to address, steps taken and by whom, resources required, any barriers identified to achieving the goals, and any adaptations made and documented to the plan during the intervention.

Semistructured interviews of approximately 30 minutes will be held at 3 intervals for each intervention wave with members of the planning ahead teams and the supporting in-reach teams. The interview guides will draw on realist interviewing, in which the intervention theory is the subject of the interview and presented to the interviewee for critique, refinement, or to propose alternate theories [66]. The IMPART research team aim to recruit 2-4 residential aged care facility staff to the planning ahead team at each participating site. As a result, using purposive sampling, the target number of interviews for each case is 6-10 interviews. In accordance with RAMESES, when sufficient evidence has been found across the cases such that it is reasonable to claim that a program theory is coherent and plausible, saturation will be considered reached for the interview sample [64].

Potential participants will be recruited by sending an interview invitation letter. Interested participants will be sent an information and consent form for review before meeting with the interviewer and given the opportunity to ask questions. Consent will be collected in written form and verbally confirmed by the interviewer before the interview commences. Interviews will be conducted and audio-recorded in-person, online, or by telephone, pending the participant’s preference. An honorarium of AUD $50 (approximately USD $33) will be offered to acknowledge their time and expertise. During transcription, all identifiable information will be removed from interview transcripts to protect participant anonymity, and a participant ID number will be used instead.

The first stage of interviews will be conducted following the participant’s attendance at the IMPART workshop. This interview will explore the participant’s motivation for joining the planning ahead team, as well as investigate the contextual factors, barriers and enablers that they anticipate may affect the implementation of IMPART at the residential aged care facility. The second stage of interviews will take place upon completion of the intervention at the residential aged care facility. The interviews will explore perceptions of the intervention outcomes, conditions that supported or hindered the implementation, and how this corresponds to the initial program theories. The final interview stage will take place approximately 3 months following the completion of the intervention at the residential aged care facility to explore participants’ views on the sustainability of the program.

#### Quantitative Data

Each site will complete a residential aged care facility information form, which will provide key demographic information about the facility, as well as their telehealth capacity and any concurrent projects or capacity-building taking place at the facility that may impact the implementation of IMPART. The residential aged care facility information will be used to describe the context in which the IMPART intervention is rolled out and to make cross-comparisons about how this may have influenced implementation at the varying sites similarly or differently.

Members of the planning ahead team and in-reach teams will complete an activity log to record telehealth consultations held during the intervention period, including consultations with the specialist palliative in-reach team for training purposes or discussions with residents or family. Other activities recorded in the activity log relating to intervention implementation include meetings, administration, and training (received or provided). At the conclusion of the intervention the activity logs will be assessed. For each site, the research team will collect data on how many and which of the IMPETUS-D online training modules were completed and by whom. Attendance at goals-of-care workshops will also be recorded.

#### Data Analysis

The preferred approach to data analysis in realist research is retroduction, which uses both inductive and deductive logic as well as insights or hunches [40]. To retroductively analyze the nonparticipant observations and interview transcripts, data will be imported into NVivo software (NVivo 14), and initial program theories from the coding framework created in the theory generation phase added as codes. A first reading of data will be undertaken to familiarize and extract demographic information. Data will be analyzed thematically using the coding framework and retroductive reasoning to form new CMOCs. Taking guidance from Gilmore et al [67] memos will be attached to coded content with the following fields: initial program theory, quote, source, context, implementation strategy, mechanism, outcome, CMOC, supports/refutes/refines, decision-making process, links to other initial program theories, and additional notes.

Findings will be organized according to case and compiled to build an evidence base whereby like CMOCs occur. The CMOCs will be reviewed for demi-regularities to form program theories for each case. The prevalence and strength of a CMOC will be indicated by how many participants expressed a similar experience [67]. Approximately 20% of the interview transcripts will be coded independently by the primary researcher and a second coder. The primary researcher will assign 1-2 transcripts at a time to the second coder, then arrange a meeting to cross-check codes and discuss for consistency. Where discrepancies occur, a senior member of the research team will be consulted. The primary researcher will then code the remaining transcripts using the same process.

The data collected from the action plans will undergo document analysis to validate findings from interviews and to support the development of individual case reports. Using inductive coding, the content of the action plan will be categorized, and causal inferences will be made about what has worked or not worked in enabling the planning ahead team to adopt its goals. These inferences will be tested against the hypotheses put forward in the initial program theories and later compared to make cross-case comparisons.

CMOCs will be linked to the quantitative data informing the evidence base for the descriptions of context and implementation outcomes. Results will assist in validating findings from interviews and support the development of individual case reports. The data collected from the residential aged care facility information form, activity logs, IMPETUS-D online training package, and goals-of-care training attendance will be analyzed with descriptive statistics. Responses from the residential aged care facility information form will be compared to identify any patterns between facilities that link context (eg, facility size, staffing, telehealth capacity) and implementation outcomes. Activity log statistics will be linked to fidelity and feasibility of the IMPART protocol (Did the planning ahead team and in-reach meet regularly as intended, and in what format?) and telehealth adoption (How frequently was telehealth used during the active intervention period?). Statistics extracted on online training engagement will provide a measurement of fidelity and adoption.

#### Phase 3: Theory Refinement

Following the theory testing phase, all CMOCs and program theories from the case studies will be reviewed for demi-regularities (frequently occurring themes or patterns) to make cross-case comparisons [40,64]. The prevalence and strength of a CMOC will be indicated by how many participants expressed a similar experience. Descriptive statistics will undergo comparative analysis to identify similarities and differences in context and implementation outcomes. Statistics will then be matched to qualitative data (context and implementation outcomes) to aid the explanation of patterns across the CMOCs. Cross-case theories will be arranged according to implementation outcomes to identify key causal mechanisms that either enable or hinder success.

The IMPART research team will be presented with the program theories and supporting evidence for discussion and will be asked to review the theories based on their own experience with implementing IMPART and to explain why they may support, refute, or refine certain theories. The key points from the discussion will be captured in field notes and checked and validated against available literature. Consent will be implied by participation in this theory refinement consultation. A refined cross-case program theory will then be finalized and reported back to the broader research team.

## Results

The RCT commenced in May 2023, with completion anticipated in November 2025. Funding began in January 2022. Data for the realist process evaluation will be collected between May 2023 and February 2026. As of October 2025, 61 interviews have been conducted. Data analysis is ongoing. Results are expected to be disseminated through publications and conference presentations in 2026.

## Discussion

### Overview

This study describes a realist process evaluation protocol as part of a pragmatic stepped-wedge cluster RCT with the aim to identify and explore the contexts and mechanisms that enable or hinder the implementation of the IMPART intervention. Realist process evaluation is increasingly being used to evaluate RCTs as a way to engage a theory-informed approach to evaluation while preserving the design strengths of a RCT [43,44,68]. By sharing this protocol, we hope to contribute to discussion around the usefulness of combining these approaches and provide learnings to other interested researchers. The refined program theory developed will link implementation strategies to contextual factors specific to residential aged care that trigger mechanisms influencing the implementation of IMPART. There is a lack of understanding of the effects of different implementation strategies and the extent to which they can facilitate the uptake of interventions, particularly in residential aged care [53,54,69-76]. Implementation strategies are underreported and difficult to differentiate from the intervention. Applying a realist framework to explore process outcomes therefore allows for implementation to be analyzed and reported in detail. This realist process evaluation is novel in offering an explanatory account of what has worked or not worked in the implementation of a palliative care intervention in residential aged care.

This study also addresses key gaps in the evaluation and implementation of telehealth use for end-of-life care in residential aged care. The findings will be timely in their congruence with national strategic priorities around digital health [24]. As telehealth expands, it is important to monitor its use in complex settings such as residential aged care and ensure it is achieving the desired outcomes for residents, their caregivers, and the residential care workforce. This will be the first realist process evaluation to uncover causal mechanisms that influence the uptake of telehealth in residential aged care to support the provision of palliative care.

Residential aged care facilities are complex sites undergoing constant change. High staff turnover, lack of staff capacity at all levels, ongoing compliance and training obligations often impacts the ability of their workforce to engage meaningfully with research [54,70,74]. While it may be challenging to engage residential aged care staff into the study, the factors impacting engagement will be considered in analysis as part of the context influencing the implementation of the IMPART intervention. Similarly, any adaptations made by the participants or research team to the trial protocol to mitigate barriers to successful implementation will be documented and reported as part of the process evaluation but will not be informed by it. Effort has been taken in the selection of methods to minimize the burden of participation on residential aged care staff as their insights are crucial to progressing the translation of research evidence into practice.

### Strengths and Limitations

Realist evaluation and synthesis is still considered a relatively new methodology, and using it comes with its own set of challenges [37]. Key to realist inquiry is flexibility to adapt the methodology to emerging theories [40,64]. Ethical approval for the trial protocol, selection of methods, and data collection materials such as interview guides is required before implementation of any intervention. This can make it difficult to undertake a truly realist-informed process evaluation within the parameters of the RCT rollout and budget timelines, whereby selection of methods and interview questions is informed by the initial program theories [40,64].

The methods for the IMPART realist process evaluation were selected before the development of the initial program theories and therefore are not truly realist-informed. It is intended that the semistructured interview format will allow for interview guides to be molded to test the initial program theories as they evolve and change with each facility. The initial program theories will be tested across up to 10 cases, allowing sufficient opportunity to present and discuss these theories using realist interviewing techniques. The coding framework applied to the interview transcripts is also informed by the initial program theories to ensure their interpretation explores the hypothesized mechanisms. While the overarching research questions are not specific to these hypothesized mechanisms, they are general enough to still reflect the evaluation aims of uncovering hidden causal linkages between context and mechanisms that lead to implementation outcomes. Nonetheless, this is an important consideration for researchers looking to integrate a realist process evaluation into an RCT.

A limitation of the realist approach is that the full extent of contexts and mechanisms influencing the implementation of the intervention is unlikely to be uncovered in one study, with findings from a realist evaluation not generalizable [37]. However, the findings from this study will provide rich insights that contribute to growing knowledge on what works, for whom, how, and under what circumstances in implementing interventions that seek to improve palliative care in residential aged care. The strength of this realist process evaluation lies in its potential to provide transferable, context-specific findings that can support the development of meaningful policy and accelerate practice change [37].

Another strength of the methodology is that it centers the voices of the residential aged care workers asked to undertake implementation activities. The findings will consequently go beyond quantitative measures of intervention adoption and implementation fidelity to integrate meaningful causal explanations with real-world implications.

### Future Directions

Findings from the trial will be communicated back to participating residential aged care facilities and stakeholders through a written project summary prepared by the research team. Results will also be disseminated to the broader research community through conference presentations and publications. If IMPART is shown to be effective by trial outcome measurements, namely reducing unplanned hospitalizations at the end of life, the refined program theory from this realist process evaluation will inform the rollout of future iterations of IMPART to the residential aged care sector (including those in regional, rural, and remote areas), continuing the cycle of theory testing and refinement key to realist inquiry. The implementation plan for future rollout of IMPART will identify key causal pathways that have led to success during the trial, which can then inform decision-making around the level of support and resources a particular facility may need. In doing so, we can move closer to ensuring residential aged care staff have access to valuable professional development opportunities to develop their confidence and competence to provide quality palliative care, and that residents have access to high-quality end-of-life care they should expect.

### Conclusions

Applying a realist framework to explore process outcomes allows for an in-depth inquiry into what works, for whom, how, and in what circumstances in the implementation of complex interventions that aim to improve palliative care in residential aged care. The refined program theory developed will help accelerate efforts to implement telehealth-enabled interventions to improve the provision of quality palliative care in residential aged care.

## Appendix

Multimedia Appendix 1 Initial Program Theories of the IMPART program.

## Abbreviations

CFIR: Consolidated Framework for Implementation Research
CMOC: context–mechanisms–outcomes configurations
COM-B: Capability, Opportunity, and Motivation inform Behavior change
ERIC: Expert Recommendations for Implementing Change
IMPART: Improving Palliative Care in Residential Aged Care Using Telehealth
IMPETUS-D: Improving Palliative care Education and Training Using Simulation in Dementia
RAMESES: Realist and Meta-narrative Evidence Syntheses: Evolving Standards
RCT: randomized controlled trial
