# Supplementary material for: Improving Palliative Care in Residential Aged Care Using Telehealth: Protocol for a Realist Process Evaluation Embedded in a Stepped-Wedge Cluster Randomized Controlled Trial
Source: JMIR Res Protoc. 2025 Oct 20;14:e68332. doi: 10.2196/68332 (PMC12583945; doi:10.2196/68332)
Supplement: Multimedia Appendix 1 [file resprot_v14i1e68332_app1.docx]

**Appendix 1 – Initial Program Theories (IPT) of the IMPART program**

**Research question:** What works, for whom, how and in what circumstances in the implementation of the IMPART (IMproving PAlliative care in Residential aged care using Telehealth) program?

Context-Mechanism-Outcome (C-M-O) configurations

| **IPT no.** | **Context (C)** | **Implementation strategy** | **Mechanism (M)** | **Implementation outcome (O)** | **Data collection measures** |
| --- | --- | --- | --- | --- | --- |
| 1. **By formalising the commitment of the recruited residential aged care facilities to the implementation of IMPART through signed agreements the support of senior residential aged care facility managers and leaders for IMPART is legitimised and initiates participation to adhere to those commitments and implement IMPART as intended.** | Senior staff & managers provide strong, supportive & engaged leadership for IMPART | *Pre-implementation:* Formal commitments obtained via signed agreement | The residential aged care facility’s participation in IMPART is legitimised and initiates action to adhere to formal commitments | Fidelity to intervention manual   - Planning Ahead Team meets fortnightly - Actions are taken to achieve goals set - Monthly telehealth consultation is held with In-Reach - Participants attend Goals of Care training - Participants complete IMPETUS-D modules | - **Semi-structured interview** - **Activity log** - **Action plan** - **Training completion** |
| 1. **By providing champions with user-friendly intervention manuals and regular reminders emailed by the research team with clear directions champions are able to make sense of the intervention protocol and track their progress leading to fidelity to the intervention manual.** | IMPART & its resources are well designed & user-friendly with information about the implementation process accessible to all champions | *Component 1:* Intervention manual provided to site champions with reminders sent by external facilitators for key tasks | Champions are able to make sense of the intervention protocol and track progress | Fidelity to intervention manual   - Planning Ahead Team meets fortnightly - Actions are taken to achieve goals set - Monthly telehealth consultation is held with In-Reach - Participants attend Goals of Care training - Participants complete IMPETUS-D modules | - **Semi-structured interviews** - **Activity log** - **Action plan** - **Training completion** |
| 1. **By instructing residential aged care facility champions to undertake a needs analysis (file audit and confidence survey) participants in the Planning Ahead Team are given the opportunity to reflect and evaluate their current end-of-life care practices and identify potential gaps that could be resolved through the implementation of IMPART thus differentiating it from current ways of working and leading to the perception among participants that IMPART fulfils a need at the residential aged care facility.** | Reflection & evaluation is embedded into the IMPART intervention | *Component 2:* Champions undertake needs analysis | Champions differentiate IMPART from current ways of working and view it as opportunity to resolve gaps identified through the needs analysis | Participants agree that IMPART meets a need and/or is a good match for their residential aged care facility (appropriateness) | - **Semi-structured interview** - **Non-participant observation** - **Action plan** |
| 1. **By engaging each Planning Ahead Team into action planning and setting site-specific goals IMPART can be adapted and tailored to the specific workflow and needs of each residential aged care facility which allows Planning Ahead Team members to collectively establish consensus leading to** **the perception among participants that IMPART fulfils a need at the residential aged care facility.** | IMPART is adaptable, flexible & can be tailored to the local context and workflow | *Component 3:* Intervention goals tailored to each residential aged care facility through action planning | Champions establish collective consensus through goal setting and reflexive monitoring of action plan | Participants agree that IMPART meets a need and/or is a good match for their residential aged care facility (appropriateness) | - **Semi-structured interview** - **Non-participant observation** - **Action plan** |
| 1. **By allocating funding from the research project budget to backfill staff participation in the implementation of IMPART the residential aged care facility is ensured access to adequate resources and opportunity for staff participation allowing the implementation of the intervention to be viewed as more feasible.** | Adequate funding is in place to ensure appropriate staffing & access to resources | *Pre-implementation:* Funds provided to backfill staff participation | Opportunity is created for staff to participate in implementing IMPART | Participants agree that implementation of IMPART is feasible | - **Semi-structured interview** - **Activity log** |
| 1. **As residential aged care facilities are known to experience high staff turnover and time limitations then building a team between 2-4 residential aged care facility champions and facilitators from the supporting In-Reach service allows for collective action and shared workload as well as mitigating potential changes to the Planning Ahead Team membership leading to increased continuity and feasibility of IMPART implementation.** | High staff turnover and time limitations | *Component 5:* Teaming by building coalition between PAT and In-Reach | The Planning Ahead Team share the workload of participation and take collective action to implement IMPART | Participants agree that implementation of IMPART is feasible | - **Semi-structured interview** - **Activity log** |
| 1. **Whereby champions identified and prepared for implementation of IMPART demonstrate motivation to improve end-of-life care practice at their residential aged care facility and are supported by a learning centred culture that is open to change and provides opportunity to participate in the intervention, then likelihood of uptake of IMPETUS-D modules and Goals of Care training are increased.** | Facility has a learning centred culture that is open to change, and staff are allocated time to participate in implementation | *Component 1:* Identify and prepare champions  *Component 4:* Provide training opportunities | COM-B | Adoption of IMPETUS-D and Goals of Care   - Completion of modules/training - Any action taken to try to employ learnings from IMPETUS-D or Goals of Care training | - **Semi-structured interview** - **Training completion** |
| 1. **Whereby participating residential aged care facilities demonstrate a learning centred culture that is open to change and view increased use of telehealth to have potential benefits to staff, residents and their families, then champions identified and prepared for the implementation of IMPART are more likely to advocate for and act as early adopters of telehealth to contact palliative care specialists.** | Facility has a learning centred culture that is open to change, and telehealth is perceived as beneficial for staff, residents and their families | *Component 1:* Identify and prepare champions | Champions advocate for and act as early adopters of telehealth to contact palliative care specialists | Adoption/uptake of specialist telehealth   - Monthly telehealth consultation is held with in-reach to discuss end-of-life care as intended - Any action taken to try to adopt the use of telehealth for rapid consultation with palliative care specialists | - **Semi-structured interview** - **Activity log** |
| 1. **Whereby timely palliative care services are available to the residential aged care facility via a local in-reach service, engaging representatives from that in-reach team to act as external facilitators supporting the Planning Ahead Team to implement impart increases the frequency of contact, familiarity and trust between the residential aged care facility and in-reach service leading to membership of in-reach team to the residential aged care facility and routinisation of consultation.** | Timely palliative care services are available | *Component 5:* External facilitation (In-Reach) and educational outreach | Regular contact with In-Reach team increases trust between services and grants the In-Reach team membership to the residential aged care facility | Intervention sustained   - Routine contact with the In-Reach service is ongoing post-intervention - Participants report an increase in trust, respect and/or comfort in working with the residential aged care facility staff/in-reach service | - **Semi-structured interview** |
